# Choroidal Detachment in a Patient on a CPAP Therapy

**DOI:** 10.1155/crop/2417779

**Published:** 2026-07-29

**Authors:** Emilia Żurowska, Emilia Zwolińska, Iwona Rospond-Kubiak

**Affiliations:** ^1^ Department of Ophthalmology, Poznań University of Medical Sciences, Poznań, Poland, ump.edu.pl

**Keywords:** choroidal detachment, continuous positive airway pressure, CPAP, obstructive sleep apnea

## Abstract

**Objective:**

The aim of the study is to present a rare case of choroidal detachment in a patient on continuous positive airway pressure (CPAP) therapy.

**Material and Method:**

A 76‐year‐old man on CPAP therapy due to obstructive sleep apnea presented to the ocular oncology clinic with a suspicion of an intraocular tumor. He complained of tunnel vision in red, floaters and flashes in the visual field, and irritation of the left eye since 13 weeks.

**Results:**

On initial assessment, the best corrected visual acuity (BCVA) of the left eye was 1.0, and the intraocular pressure (IOP) was 21 mmHg. The examination revealed dilated, tortuous episcleral vessels and a massive choroidal effusion. The patient was diagnosed with choroidal detachment and was treated with topical dexamethasone 0.1% and cyclopentolate. On a follow‐up visit 3 weeks later, the BCVA got worse to hand movements and IOP increased to 27 mmHg. The patient received a combination of dorzolamide and timolol in addition to the aforementioned treatment. Over the following 16 weeks, a gradual resolution of the choroidal detachment was documented.

**Conclusions:**

The CPAP treatment is a possible, though unproven, contributor to the choroidal detachment. The patient improved during corticosteroid, cycloplegic, and IOP‐lowering treatment.

## 1. Introduction

Obstructive sleep apnea (OSA) is a condition characterized by repetitive episodes of incomplete or complete upper airway obstruction during sleep [1]. The treatment of choice for this condition is the use of continuous positive airway pressure (CPAP), which relieves the blockage by delivering pressurized air using a flow generator and a mask [2]. Ophthalmological complications reported in the literature include dry eye syndrome, superficial punctate keratitis, bacterial infections, and increased intraocular pressure (IOP) [3–6].

The aim of this study is to present a rare case of choroidal detachment in a patient on CPAP therapy.

## 2. Case Presentation

A 76‐year‐old man presented to the ophthalmology outpatient clinic because of a dark lesion found upon dilated fundus examination of the left eye. He complained of tunnel vision in red, floaters and photopsias in the visual field, and redness of the affected eye. The symptoms had persisted for approximately 13 weeks. He had been previously treated for unilateral conjunctivitis with no improvement.

The patient had a history of OSA syndrome on CPAP therapy since 8 years. The CPAP therapeutic pressure was increased 20 weeks before from 4 to 5.5 cmH_2_O. At the same time, the patient was fitted with a new oronasal (full‐face) mask. No supplemental oxygen was used. The changes resulted in major improvement in time with large leak, percentage of time with large leak, and average leak rate (Table 1). The other general comorbidities were hypertension, angina pectoris, atrial fibrillation, type 2 diabetes, hyperlipidemia, and obesity.

**Table 1:** CPAP parameters over time.

Parameter	September 14, 2022	October 28, 2024
Device	Philips A‐Flex (Auto‐CPAP)	Philips A‐Flex (Auto‐CPAP)
Apnea–hypopnea index (AHI)	3.8 events/h	4.0 events/h
90th percentile therapeutic pressure	4.5 cmH_2_O	5.5 cmH_2_O
Mean pressure	4.1 cmH_2_O	4.6 cmH_2_O
Time with large leak	195 min	1.0 min
Percentage of time with large leak	47.6%	0.2%
Average leak rate	58.0 L/min	20.8 L/min
Oronasal (full‐face) mask size	L	M

**Figure 1:**
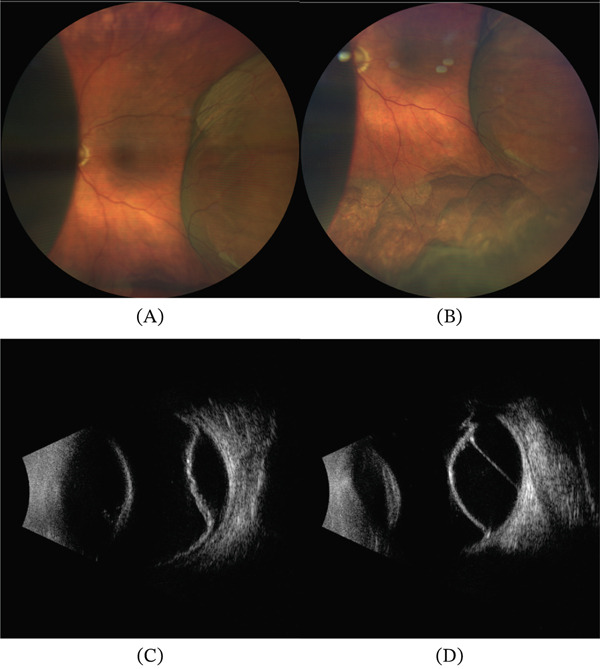
(A, B) Color fundus photograph of the left eye upon initial assessment–choroidal detachment. (C, D) B‐scan of the left eye upon initial assessment.

On initial assessment, the best corrected visual acuity (BCVA) of both eyes was 1.0, and the IOP was within normal limits. Autorefraction revealed a refractive error of +2.25 −0.75 × 90° in the right eye and +1.25 −0.75 × 13° in the left eye. The anterior segment examination of the left eye revealed dilated tortuous episcleral vessels, and a massive choroidal detachment was noted on fundoscopy (Figure 1). Based on the ocular ultrasound imaging, the presence of an intraocular tumor was excluded; however, anterior rotation of the ciliary body and forward displacement of the iris diaphragm with subsequent shallowing of the anterior chamber and narrowing of the angle could be noted on the ultrasound biomicroscopy (UBM). Apart from incipient cataract, the right eye appeared normal on anterior and posterior segment examination.

The patient was sent for laboratory tests and a contrast‐enhanced computed tomography (CT) scan of the head. He received topical dexamethasone 0.1% and cyclopentolate. The laboratory findings were unremarkable (Table 2). The CT scan excluded the presence of any intracranial hemorrhage and pathological masses.

**Table 2:** Laboratory findings at initial presentation.

Parameter	Result	Reference range
Erythrocyte sedimentation rate (ESR)	12 mm/h	1–10 mm/h
Activated partial thromboplastin time (aPTT)	24.9 s	25.4–36.9 s
D‐dimer	351 ng/mL FEU	< 500 ng/mL FEU
Alanine aminotransferase (ALT)	25 U/L	< 45 U/L
Aspartate aminotransferase (AST)	22 U/L	5–34 U/L
C‐reactive protein (CRP)	0.7 mg/L	< 5.0 mg/L
Serum creatinine	0.85 mg/dL	0.72–1.25 mg/dL
Estimated glomerular filtration rate (eGFR)	> 60 mL/min/1.73 m^2^	> 60 mL/min/1.73 m^2^
Prothrombin activity	95%	80%–120%
Prothrombin time (PT)	11.8 s	10.2–12.9 s
International normalized ratio (INR)	1.02	0.8–1.2
White blood cell count (WBC)	8.14 × 10^9^/L	4.00–10.00 × 10^9^/L
Absolute neutrophil count	4.73 × 10^9^/L	1.80–7.00 × 10^9^/L
Absolute lymphocyte count	2.58 × 10^9^/L	0.80–4.50 × 10^9^/L
Absolute monocyte count	0.66 × 10^9^/L	0.00–0.90 × 10^9^/L
Absolute eosinophil count	0.11 × 10^9^/L	0.00–0.50 × 10^9^/L
Absolute basophil count	0.04 × 10^9^/L	0.00–0.15 × 10^9^/L
Immature granulocyte count	0.02 × 10^9^/L	0.00–0.04 × 10^9^/L
Neutrophils	58.1%	45.0%–70.0%
Lymphocytes	31.7%	20.0%–45.0%
Monocytes	8.1%	0.0%–9.0%
Eosinophils	1.4%	0.0%–5.0%
Basophils	0.5%	0.0%–1.5%
Immature granulocytes	0.2%	0.0%–0.4%
Red blood cell count (RBC)	4.66 × 10^12^/L	4.50–5.50 × 10^12^/L
Hemoglobin (Hb)	9.6 mmol/L	8.0–11.0 mmol/L
Hematocrit (Hct)	0.46 L/L	0.40–0.54 L/L
Mean corpuscular volume (MCV)	97.9 fL	82.0–97.0 fL
Mean corpuscular hemoglobin (MCH)	2.06 fmol	1.64–2.08 fmol
Mean corpuscular hemoglobin concentration (MCHC)	21.1 mmol/L	20.0–22.0 mmol/L
Red cell distribution width (RDW‐CV)	12.4%	11.0%–15.0%
Platelet count (PLT)	138 × 10^9^/L	130–390 × 10^9^/L
Mean platelet volume (MPV)	10.7 fL	7.0–11.0 fL
Platelet distribution width (PDW)	12.6 fL	9.0–17.0 fL

On a follow‐up visit 3 weeks later, a decrease in visual acuity in the left eye and the progression of the choroidal effusion were noted. The BCVA of the left eye dropped to hand movements, and the IOP was elevated to 27 mmHg. At this stage, the patient was diagnosed with choroidal effusion of the left eye following CPAP treatment. The ongoing treatment was supplemented by dorzolamide and timolol combination, and the patient was referred for further consultation to his pulmonologist. He confirmed the current CPAP test readings were consistent with the required standards, and due to pulmonological indications, continuing with the therapy was advised. Additionally, the patient underwent rheumatologic evaluation, which did not identify evidence of an underlying systemic inflammatory or rheumatologic disease associated with choroidal detachment.

**Figure 2:**
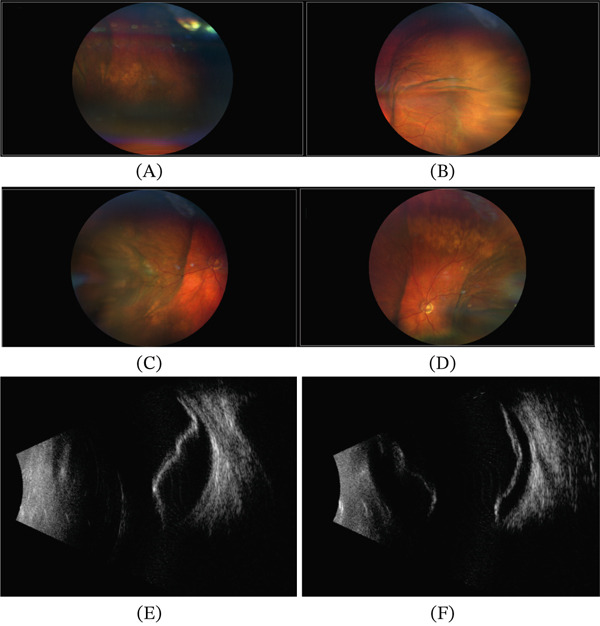
(A, B, C, D) Color fundus photograph of the left eye at 7‐week follow‐up after treatment initiation. (E, F) USG B of the left eye at 7‐week follow‐up after treatment initiation.

At the next follow‐up visit after 4 weeks, the patient noted subjective improvement in the visual acuity of the left eye. The BCVA of the left eye was 0.2 and IOP stabilized at 17 mmHg. The episcleral vasodilation and choroidal effusion partly resolved (Figure 2). The current treatment was maintained.

**Figure 3:**
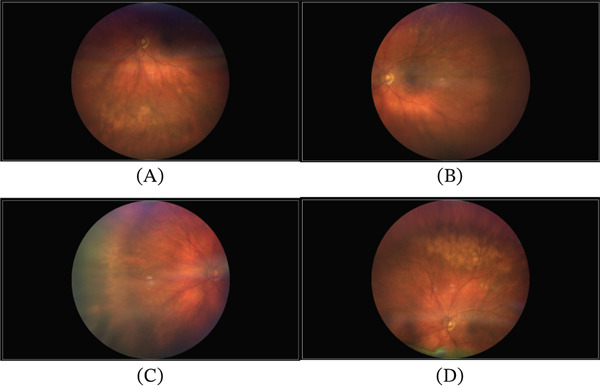
(A, B, C, D) Color fundus photograph of the left eye at 16‐week follow‐up after treatment initiation.

Over the following 9 weeks, a gradual resolution of the choroidal detachment was observed (Figure 3). The medications regimen was adjusted as required along the course of treatment. Eventually, dexamethasone was gradually reduced and discontinued. Cyclopentolate was withdrawn, and the dorzolamide and timolol combination was replaced with brimonidine. On the most recent appointment, 18 weeks from the initial visit, the BCVA of the left eye was 0.4, and the IOP was 16 mmHg. The configuration of the ciliary body and the iris returned to a normal position, and there was no choroidal detachment. However, a progression of a lens opacification in the left eye was noted, and the patient was qualified for cataract surgery.

A CARE‐compliant timeline summarizing the chronology of events is presented in Table 3.

**Table 3:** CARE‐compliant timeline of clinical presentation, treatment, and follow‐up.

Time point	Clinical course and key findings	BCVA of the left eye	IOP (mmHg) of the left eye	Management
Approximately 20 weeks before presentation	Change in CPAP parameters and mask (suspected precipitating factor).	—	—	—
Approximately 13 weeks before presentation	Symptom onset: red‐tinted tunnel vision, floaters, photopsias, and ocular redness of the left eye.	—	—	—
Baseline	Initial ophthalmic evaluation. Dilated episcleral vessels, anterior rotation of the ciliary body, forward displacement of the iris–lens diaphragm, shallowing of the anterior chamber, angle narrowing, and choroidal detachment	1.0	Normal	Initiated topical dexamethasone and cyclopentolate. Laboratory investigations and contrast‐enhanced CT ordered.
Week 3	Progression to kissing choroidal detachment.	Hand movements	27	Added topical dorzolamide/timolol. Continued dexamethasone and cyclopentolate. Pulmonology consultation confirmed appropriate CPAP parameters. Rheumatology evaluation did not reveal evidence of an underlying theumatological disorder.
Week 7	Partial resolution of episcleral vessel dilation and choroidal effusion.	0.2	17	Continued treatment.
Week 16	Complete anatomical resolution of choroidal detachment. Further improvement in episcleral vessel dilation, ciliary body position, and iris configuration.	0.2	28∗	Discontinued cyclopentolate. Began tapering dexamethasone. Continued dorzolamide/timolol.
Week 18	Stable anatomical findings with only mild residual episcleral vessel dilation. Progressive cataract noted.	0.4	16	Continued follow‐up, further tapering and discontinuation of dexamethasone; qualified for cataract surgery.

*Note:* Asterisk “∗” denotes the patient reported not using topical IOP‐lowering medication on the morning of the visit.

## 3. Discussion

This is a report of a rare case of choroidal detachment as a possible complication of CPAP therapy due to OSA. The diagnosis was made based on ocular imaging and the exclusion of other ophthalmic and general causes of choroidal detachment.

According to the literature, other risk factors for choroidal detachment include hypotony following surgical procedures, ocular trauma, various ocular inflammatory diseases, glaucoma, nanophthalmos, Sturge–Weber syndrome, and the use of certain medications, notably IOP‐lowering agents. Additionally, nonophthalmic conditions that have been identified as contributing factors comprise hypertension (especially hypertensive crisis and malignant hypertension), end‐stage renal disease, toxemia of pregnancy, and pheochromocytoma [7–13].

The differential diagnosis included nanophthtalmos and uveal effusion syndrome, as both conditions may be associated with choroidal detachment. However, neither ultrasonography nor UBM showed other features typically associated with these entities, such as scleral thickening or retinal detachment. Additionally, the patient was only mildly hyperopic, and the clinical course was transient with complete anatomical recovery, which is not a characteristic of these syndromes [14–16].

The likelihood of thyroid‐associated orbitopathy was considered low, as the patient had neither history nor clinical manifestations of thyroid disease. Ophthalmological examination did not reveal typical features of thyroid eye disease, such as eyelid retraction, proptosis, periorbital edema, restrictive ophthalmoplegia, or conjunctival chemosis [17]. Therefore, thyroid function testing was not performed. However, thyroid‐associated orbitopathy cannot be formally excluded and should be acknowledged as a diagnostic limitation.

Despite the patient′s history of hypertension, the disease was effectively managed. The fundus examination revealed no evidence of hypertensive chorioretinopathy as indicated by the absence of arteriolar narrowing, arteriovenous nicking, cotton wool spots, hemorrhages, disk swelling, or macular edema in any eye.

Although contrast‐enhanced CT was unremarkable, the absence of magnetic resonance imaging/magnetic resonance angiography or digital subtraction angiography represents a limitation, as these modalities are more sensitive for detecting low‐flow carotid‐cavernous fistulas, dural arteriovenous fistulas, and subtle cavernous abnormalities [18].

The increase in outflow pressure on his CPAP machine, together with the introduction of a new oronasal mask occurred shortly before the onset of the symptoms. Notably, the change in mask was associated with marked improvements in therapy delivery, as reflected by reductions in time with large leak, percentage of time with large leak, and average leak rate. Although the increase in the pressure was relatively small, the improved mask fit resulted in a substantially greater effective pressure exposure. In the absence of compelling evidence to suggest that the systemic condition was responsible for the choroidal detachment, it has been proposed that the patient developed it as a complication of the CPAP therapy.

The exact pathomechanism leading to a choroidal detachment during CPAP therapy is not quite clear. The available literature has not provided a definitive explanation for this complication. Although further studies are warranted, the rarity of the condition and the limited number of reported cases hamper such investigations. Our hypothesis is that the increase in effective CPAP pressure following adjustment of the pressure and a marked reduction in mask leak after the introduction of a new oronasal mask 20 weeks prior to the first consultation in our clinic resulted in reduced venous drainage from the choroid. Available literature does not directly link CPAP therapy with episcleral venous pressure. However, the choroid drains through the vortex veins into the ophthalmic venous system, and elevations in central venous pressure may be transmitted to the choroidal circulation. Positive airway pressure increases intrathoracic pressure and may reduce venous return, which can be appreciated during a Valsalva maneuver [19]. During a Valsalva maneuver, the increase in airway pressure has been associated with increases of retinal venous pressure and IOP [20, 21]. Extrapolating from these observations, CPAP therapy may similarly impair choroidal venous drainage, resulting in venous congestion, increased choroidal hydrostatic pressure, accumulation of fluid within the suprachoroidal space, and the detachment of the choroid. As no intervention was made, the condition progressed to the ciliary body detachment and iris anterior rotation. This, in turn, caused narrowing of the angle and elevated IOP.

In the case we present, the patient improved during topical therapy using dexamethasone, cyclopentolate, thymolol, and dorzolamide. Compared with the case described by Simaroj and Preechawat [22], the duration of the treatment was 16 weeks longer (18 weeks in total). The CPAP therapy in our patient was maintained, as opposed to that of the other patient and, based on the ultrasound imaging available, the degree of the choroidal detachment was more extensive. Additionally, the therapy consisted of initially two and later four medications rather than dexamethasone alone.

The visual acuity course reflected the anatomical changes observed during follow‐up visits. Despite treatment initiation at first presentation, at the beginning the choroidal detachment progressed to kissing configuration involving the macular visual axis, which was accompanied by a decline in BCVA of the left eye from 1.0 to hand movements. Subsequent regression of the detachment resulted in gradual visual recovery to 0.2 and later 0.4 during follow‐up. However, regardless of complete anatomical resolution by the third month of therapy, BCVA did not return to its baseline value of 1.0. This may suggest that lenticular opacity became a factor limiting visual function after resolution of the choroidal detachment and a reason for the discrepancy between anatomical and functional outcomes.

In conclusion, we believe that a careful ophthalmological assessment should always be advised if the patient is undergoing the CPAP therapy. We hope that this report will contribute to raising awareness of the choroidal detachment as the potential complication of the treatment.

## Funding

No funding was received for this manuscript.

## Consent

Written informed consent was obtained from the patient for publication of this case report and any accompanying clinical images. A copy of the written consent is available for review by the editor‐in‐chief of this journal upon reasonable request.

## Conflicts of Interest

The authors declare no conflicts of interest.

## Data Availability

The data that support the findings of this study are available on request from the corresponding author. The data are not publicly available due to privacy or ethical restrictions.
